# Amplification of the Melanocortin-1 Receptor in Nephrotic Syndrome Identifies a Target for Podocyte Cytoskeleton Stabilization

**DOI:** 10.1038/s41598-018-34004-7

**Published:** 2018-10-24

**Authors:** Lovisa Bergwall, Hanna Wallentin, Johannes Elvin, Peidi Liu, Roberto Boi, Carina Sihlbom, Kyle Hayes, Dale Wright, Börje Haraldsson, Jenny Nyström, Lisa Buvall

**Affiliations:** 1 Department of Physiology, Institute of Neuroscience and Physiology, University of Gothenburg, Gothenburg, Sweden; 2 Department of Molecular and Clinical Medicine, University of Gothenburg, Gothenburg, Sweden; 3 The Proteomics Core Facility at the Institute of Medicine at the Sahlgrenska Academy, University of Gothenburg, Gothenburg, Sweden; 4 Mallinckrodt Pharmaceuticals, Hazelwood, Missouri USA

## Abstract

The melanocortin-1 receptor (MC1R) in podocytes has been suggested as the mediator of the ACTH renoprotective effect in patients with nephrotic syndrome with the mechanism of action beeing stabilization of the podocyte actin cytoskeleton. To understand how melanocortin receptors are regulated in nephrotic syndrome and how they are involved in restoration of filtration barrier function, melanocortin receptor expression was evaluated in patients and a rat model of nephrotic syndrome in combination with cell culture analysis. Phosphoproteomics was applied and identified MC1R pathways confirmed using biochemical analysis. We found that glomerular MC1R expression was increased in nephrotic syndrome, both in humans and in a rat model. A MC1R agonist protected podocytes from protamine sulfate induced stress fiber loss with the top ranked phoshoproteomic MC1R activated pathway beeing *actin cytoskeleton signaling*. Actin stabilization through the MC1R consisted of ERK1/2 dependent phosphorylation and inactivation of EGFR signaling with stabilization of synaptopodin and stressfibers in podocytes. These results further explain how patients with nephrotic syndrome show responsiveness to MC1R receptor activation by decreasing EGFR signaling and as a consequence restore filtration barrier function by stabilizing the podocyte actin cytoskeleton.

## Introduction

Membranous glomerular nephropathy (MN) and Focal Segmental Glomerulosclerosis (FSGS) are the most common primary causes of nephrotic syndrome. There has been a lack of efficient treatment for these patients but in 1999, Berg *et al*. reported on patients with MN which were treated with ACTH in an attempt to lower blood cholesterol levels^1^. As an unexpected consequence, the ACTH treatment was found to have beneficial effects on glomerular function, such as increased glomerular filtration rate (GFR) and reduced proteinuria. Several successful studies followed wherein ACTH has been administered to steroid- and immunosuppressant-resistant patients with glomerular diseases affecting the podocytes, such as MN and FSGS^2–4^.

When investigating the mechanisms behind the beneficial effects of ACTH treatment on podocytes we identified one of the ACTH receptors, the melanocortin 1 receptor (MC1R), to be the most abundantly expressed melanocortin receptor in glomerular podocytes^5^. Treating rats with Passive Heymann nephritis (PHN, a form of experimental MN) with specific MC1R agonists decreased urinary markers of oxidative stress, improved glomerular morphology with restoration of podocyte foot process structure and reduced proteinuria^5^.

The maintenance of podocyte foot process structure and glomerular barrier function greatly depend on spatial and temporal regulation of the actin cytoskeleton. Proteins regulating actin dynamics in podocytes have been shown to play a major role in the development of nephrotic syndrome. Mutations have been identified in actin regulatory proteins in patients with nephrotic syndrome, such as the slit diaphragm protein nephrin^6^, the ion channel TRPC6^7^ and the actin-binding protein alpha actinin-4^8^. Promising therapeutic targets in glomerular disease and actin cytoskeleton regulation are transmembrane proteins, such as β1-integrins^9^, Nephrin^6^, PLA_2_R^10^ and the transient receptor potential canonical (TRPC) ion channels TRPC5^11^ and TRPC6^7^. Furthermore, the importance of regulation of the master actin regulatory proteins, the RhoGTPases consisting of RhoA, Rac1 and Cdc42, in maintenance of podocyte function, has been demonstrated in studies showing that onset of proteinuria can be caused by activation of Cdc42 and Rac1 signaling and concomitant decrease of RhoA signaling in podocytes^11,12^. Several transmembrane receptors have shown to regulate RhoGTPase balance in podocytes. First, we showed that activation of the epidermal growth factor receptor (EGFR) resulted in increase of Rac1 and loss of stressfibers in podocytes^13^. Secondly, we showed stress fiber protection from puromycin aminonucleoside (PAN) damage by MC1R activation resulting in regulation of the redox status of the cells and induction of stressfibers through RhoA^14^.

In this study, we investigate MC1R regulation in patients and rats with nephrotic syndrome in combination with an unbiased approach to identify MC1R signaling pathways using phosphoproteomics. We show that MC1R is the ACTH receptor being augmented in nephrotic syndrome, thereby promoting restoration of stressfibers in podocytes by inhibition of EGFR signaling.

## Results

### Increased glomerular expression of MC1R in patients with FSGS and MN

**Figure 1:**
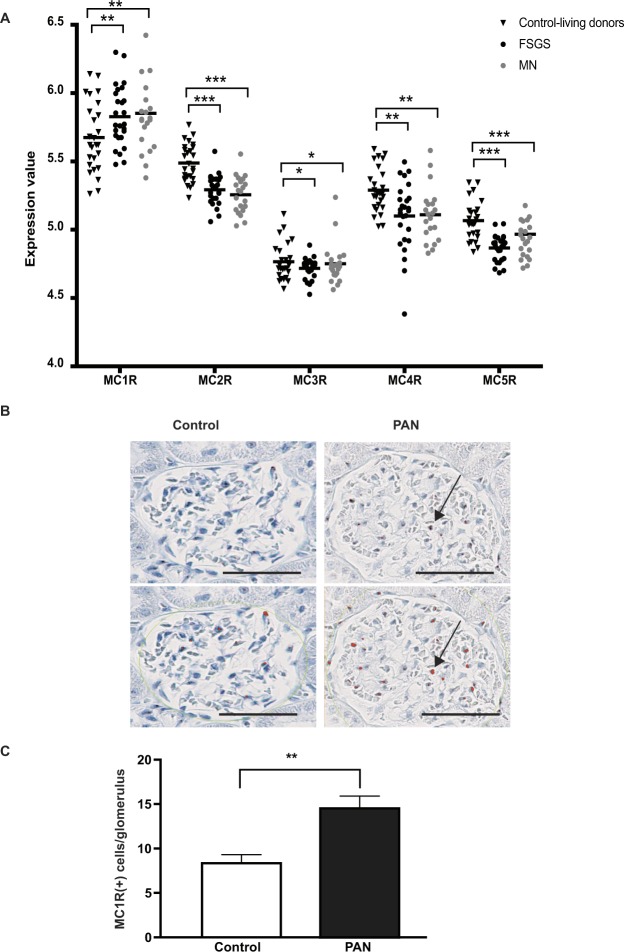
Glomerular damage increases MC1R expression. (**A**) Microarray expression data of the melanocortin receptors 1–5 (MCR) from glomeruli of FSGS and MN patients compared to healthy control-living donors. Significant Analysis of Microarray (SAM) with horizontal line representing mean value, *P < 0.5, **P < 0.01, ***P < 0.001). (**B**) Insitu hybridized MC1R in control and PAN treated rats (upper panel) showing positive cells identified by the Visiopharm quantitative image analysis software, highlighted in orange. (**C**) Positive glomerular MC1R *in situ* hybridized cells/ glomerulus in control (n = 3) and PAN treated rats (n = 8), (unpaired two-tailed t-test, **p < 0.01.). Scale bar 50 µm.

To evaluate ACTH receptor expression in nephrotic syndrome, glomerular microarray data from healthy controls and patients with glomerular diseases such as FSGS and MN were analyzed for MCR expression. 29 FSGS, 21 MN and 31 living donor control samples were used in the analysis. The groups were age and sex matched with clinical parameters GFR and mean blood pressure as shown in Table 1. All melanocortin receptors were analyzed with MC1R being the only significantly upregulated receptor while the other MCRs (2–5) were attenuated, both in patients with FSGS and MN, when compared to healthy controls, Fig. 1A.

**Table 1:** Clinical parameters of patient cohorts.

	Age	Sex (M/F)	Mean Blood Pressure (mm Hg)	GFR (ml/min/1.73 m2)	Serum Creatinine (mg/dl)
Healthy controls (n = 13)	45 ± 18	8/5	N/A	107 ± 38	0,87 ± 0,31
FSGS (n = 21)	45 ± 16	12/9	102 ± 14	73 ± 36	1,37 ± 0,90
MN (n = 21)	53 ± 18	12/9	101 ± 13	85 ± 40	1,05 ± 0,43

Data is presented as mean ± SD. In the final data analysis, another set of patients and controls (pre-transplant controls) were added that did not have any clinical data recorded, 13 healthy controls and five FSGS patients. Resulting in 26 controls, 26 FSGS and 21 MN patients used for microarray data processing.

### Increased glomerular expression of MC1R in PAN rats

To further test if impairment of podocytes gives rise to increased MC1R expression, we used a nephrotic rat, the puromycin aminonucleoside (PAN) model, which displays effaced podocytes similar to FSGS and MN. The PAN treated rats developed proteinuria by day-7, peaking at day-28 with total 24 h urinary protein of 605 ± 146 mg compared to 4 ± 0.9 mg for controls (mean ± SD). To test for glomerular MC1R expression, *in situ* MC1R hybridization with probes binding to the MC1R was performed. The rats showed a significant increase in glomerular expression of MC1R following PAN administration when compared to controls (Fig. 1B). Hybridization was visualized and analyzed using the Visiopharm software to count the numbers of positive MC1R hybridized cells per glomerulus (Fig. 1C). This further supports the view that glomerular expression of MC1R increases in renal glomerular diseases affecting the podocytes.

### Impaired podocytes increase MC1R expression

**Figure 2:**
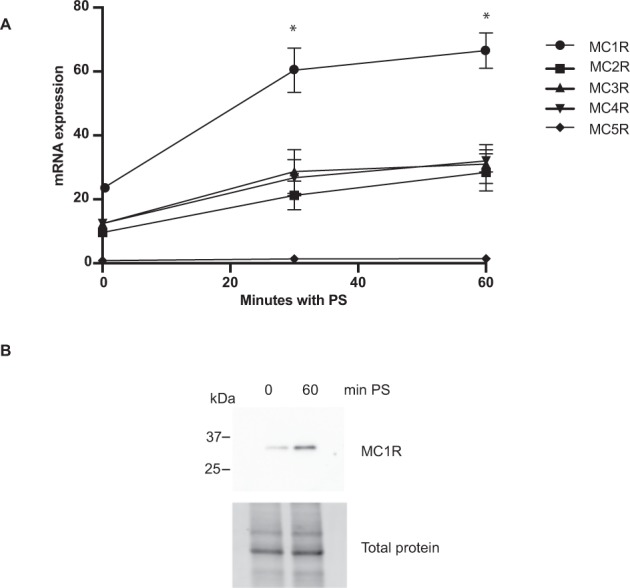
Protamine sulfate induced podocyte injury increases MC1R expression. (**A**) mRNA expression of melanocortinreceptor 1–5 (MCR) following protamine sulfate (PS) exposure. Increased MC1R mRNA expression is observed after 30 and 60 min of 600 ug/ml PS when compared to non-treated podocytes (n = 3, Students T-test, *P < 0.05), neither of the other MCRs (2–5) are significantly regulated by PS. (**B**) Protein expression of MC1R was upregulated at 60 min PS showing total protein as loading control (n = 3).

Following the microarray and nephrotic rat model analysis, there was strong evidence of amplification of MC1R expression in glomerular damage. The next step was to investigate how MC1R expression is regulated in acute podocyte damage. We used the strong cation compound, protamine sulfate (PS), that is known to damage podocytes both in cultured podocytes^13,15^ by rapid rearrangement of the actin cytoskeleton and in perfused rat kidneys by foot process effacement^15–18^. To test this, we exposed cultured podocytes to PS and analysed MCR mRNA expression. After 30 min, PS significantly increased MC1R expression peaking at termination of the experiment at 60 min, while the other MCRs (2–5) showed no significant change in expression (Fig. 2A). In addition, PS increased protein MC1R expression as shown by the western blot data in Fig. 2B. This provides further proof that podocytes specifically increase their MC1R expression in response to injury.

### The MC1R protects podocytes from protamine sulfate induced actin reorganization

**Figure 3:**
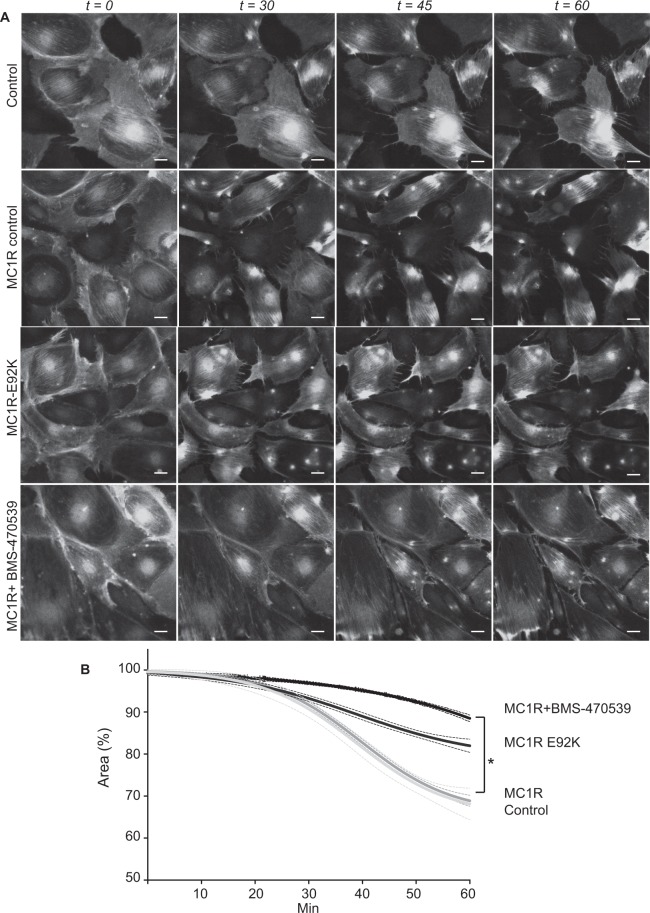
MC1R protects podocytes from actin disassembly induced by protamine sulfate. Experiments to analyze actin cytoskeleton formation were performed using LifeAct^®^, which is a 17-amino acid peptide sequence fused to GFP which binds selectively to F-actin^32^. The experiments were performed on wild type (wt) podocytes, podocytes overexpressing wt MC1R or a constitutively active MC1R mutant (MC1R-E92K)^22^. To confirm the functionality of the overexpressed MC1Rs, cAMP assays were performed following BMS-470539 treatment (Supplement Fig. 1). (**A**) Protamine sulfate (PS) induced actin cytoskeletal reorganization in cultured podocytes presented as time-lapse micrographs during treatment with PS. Representative images ofpodocytes are shown at baseline and during treatment with PS 600 µg/ml for 15, 30, 45 and 60 min. Podocytes overexpression mCherry (control), overexpressing MC1R treated with vehicle (MC1R control), MC1R mutant E92K overexpressing podocytes (E92K) and podocytes overexpressing MC1R treated with 10 nM BMS-470539 for 1 h prior to addition of PS (MC1R + BMS-470539 1 h). (**B**) Quantification of the relative podocyte area during exposure to PS using the visiopharm software (n = 3). The analyzed areas are presented over time. Scale bar 20 µm.

In a previous study, we found that MC1R promotes increases stressfibers in podocytes and protects from PAN damage by activating RhoA^14^. PAN damage is a slow process (i.e. days) leading to actin cytoskeleton rearrangement while PS damage is rapid and occurs within an hour. This suggests different mechanisms of action when it comes to cytoskeleton rearrangement. To study acute PS damage on stressfibers in real time, LifeAct^®^ studies were performed. Overexpressing wild type (wt) MC1R did not protect podocytes from PS induced stress fiber loss, while overexpressing the mutant MC1R, E92K, showed a partial rescue of stressfibers. The most prominent rescue and stabilization of podocyte morphology was found in podocytes overexpressing wt MC1R and receiving treatment with the MC1R agonist BMS-470539, as seen in Fig. 3A. Calculations of podocyte surface area showed that BMS-470539 treated MC1R overexpressing cells preserved their shape following PS exposure (Fig. 3B). These data indicate that MC1R maintains actin cytoskeleton and podocyte shape, adding an additional cytoskeleton protective effect downstream of the MC1R that acts in acute damage caused by PS.

### Identification of actin cytoskeleton pathways downstream of the MC1R

**Figure 4:**
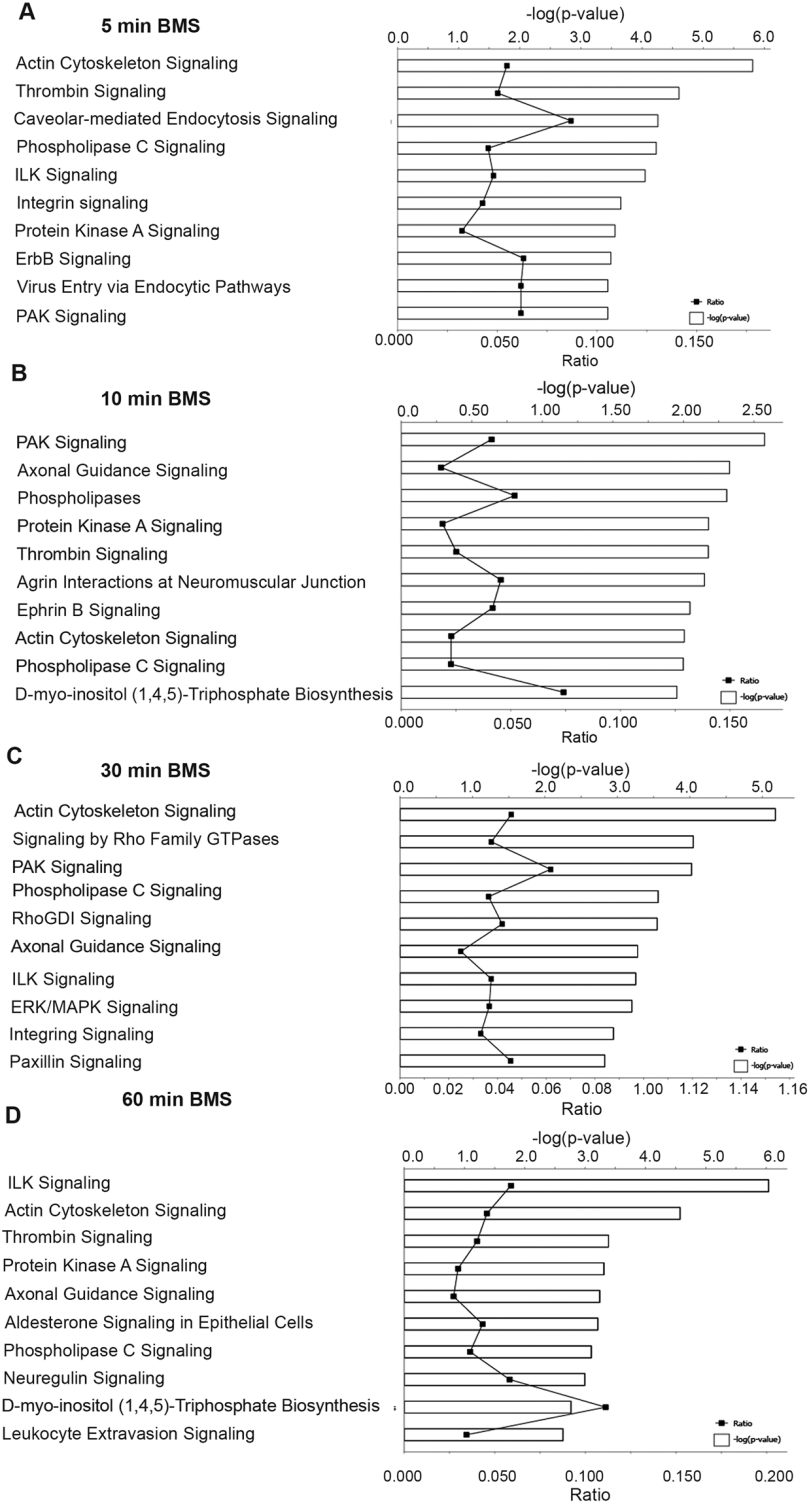
MC1R phosphorylated signaling pathways. The ten highest significantly regulated phosphorylated signaling pathways identified by Ingenuity Pathway Analysis (IPA) in MC1R overexpressing podocytes stimulated with 10 nM BMS-470539 for (**A**) 5 min, (**B**) 10 min, (**C**) 30 min and (**D**) 60 min. The bars express the −log (p-value) for the pathway. The dots shown on the lower axis represent identified proteins/total proteins in the pathway. The signaling pathways were generated through the use of IPA (QIAGEN Inc., https://www.qiagenbioinformatics.com/products/ingenuity-pathway-analysis) by using the algorithms previously described by Kramer *et al*.^29^.

**Figure 5:**
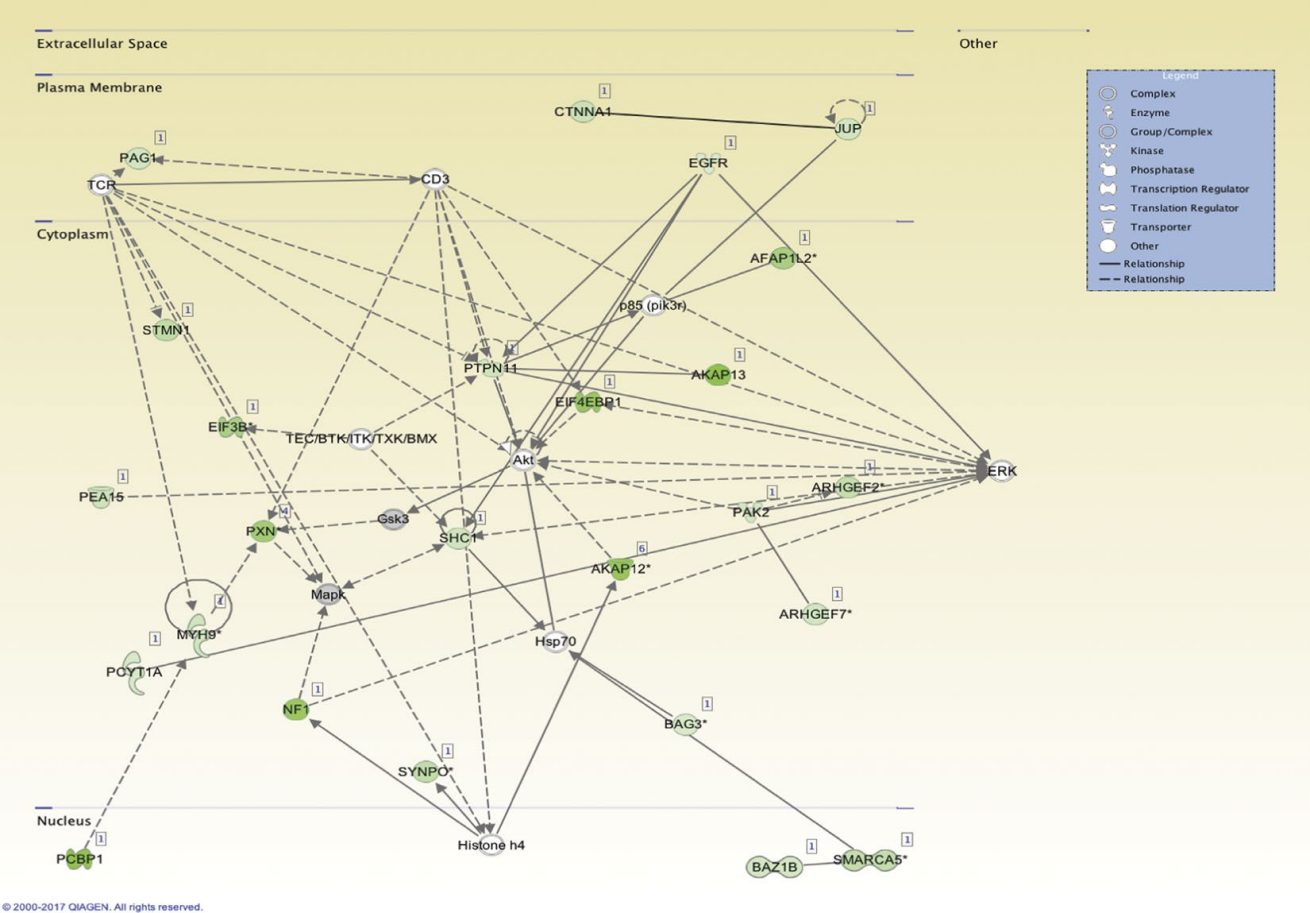
MC1R top ranked phosphorylated network at 5 min BMS-470539. The top ranked phosphorylated networks identified by IPA analysis after 5 min of BMS-470539 treatment of MC1R overexpressing podocytes. Green labeled proteins indicate that at least one peptide from that protein has been identified to be significantly phosphorylated when compared to vehicle treated controls. *More than two significantly phosphorylated peptides. The networks analyses were generated through the use of IPA (QIAGEN Inc., https://www.qiagenbioinformatics.com/products/ingenuity-pathway-analysis) by using the algorithms previously described by Kramer *et al*.^29^.

To further understand the MC1R pathways involved in actin signaling, podocytes overexpressing the MC1R treated with BMS-470539 at different time points were analyzed with phopshoproteomics. By identifying regulated phosphoproteins and integrating them into biological networks, we confirmed that MC1R stimulation is involved in pathways directly regulating actin cytoskeletal dynamics. The 10 most significantly regulated pathways at 5 to 60 min of BMS-470539 treatment are presented in Fig. 4. After 5 min of BMS-470539 treatment the top ranked regulated pathway identified was *actin cytoskeleton signaling* and in addition the interlinked pathway *integrin signaling*. In addition, regulation of the *protein kinase A pathway* indicated establishment of MC1R activation (Fig. 4A). After 10 min (Fig. 4B) the top ranked pathways was associated with RhoGTPase and actin cytoskeleton regulation, such as PAK, axonal guidance and actin cytoskeleton with the identified phospho proteins seen in supplement table 4. After 30 min of BMS-470539, RhoGTPase signaling intensified with half of the topped ranked pathways now being directly linked to RhoGTPase regulation (actin cytoskeleton, Rho Family GTPases, RhoGDI, axonal guidance, integrin and paxillin). In parallel ERK/MAPK signaling was identified to be one of the top ranked regulated pathways. After 60 min of BMS-470539 (Fig. 4D) both integrin represented by integrin-linked protein kinase (ILK) and actin cytoskeleton signaling was active. These data indicate that MC1R activation stimulates several pathways connected to actin regulation. To further understand MC1R signaling pathways, network analysis was perfomed to connect several of the identified pathways. Focusing on early signaling events, the top ranked signaling network at 5 min identified RhoGTPase regulatory Guanine Exchange factors (ARHGEF2 and ARHGEF7) together with EGFR, MAPK and synaptopodin (SYNPO), Fig. 5. Since activation of EGFR in podocytes results in destabilization of synaptopodin and loss of stressfibers by activation of Rac1 and inactivation of RhoA activity^13^, this promted us to test if MC1R is involved in stabilizing actin cytoskeleton signaling by regulating EGFR activity.

### MC1R activation of ERK 1/2 is crucial in stress fiber stabilization

**Figure 6:**
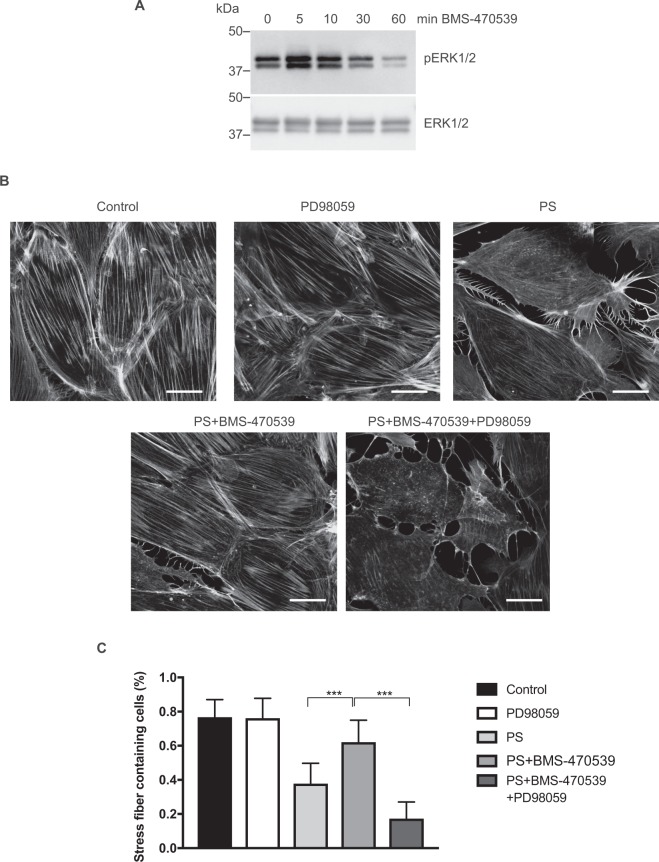
MC1R protection of actin stressfibers are promoted through ERK 1/2. The importance of ERK 1/2 in stress fiber stabilization downstream of the MC1R in podocytes was demonstrated using an inhibitor of ERK 1/2 (PD98059) in PS exposed podocytes treated with BMS-470539. (**A**) Western blots showing phosphorylated ERK 1/2 and total ERK1/2 in MC1R overexpressing podocytes treated with 0.5, 10, 30 and 60 min of BMS-470539. (**B**) Actin stressfibers visualized using phalloidin. (**C**) Actin stressfibers counted in podocytes exposed for 1 h to vehicle (control), ERK 1/2 inhibitor (PD98059), 1 h Protamine sulfate 600 ug/ml (PS), 1 h Protamine sulfate and MC1R agonist (PS + BMS-470539) and 1 h Protamine sulfate, MC1R agonist and ERK 1/2 inhibitor (PS + BMS-470539 + PD98059). The ERK 1/2 inhibitor alone did not affect stressfibers when compared to control cells. (n = 3, ***p < 0.001, ANOVA.) Scale bar 20 µm.

Activation of the MC1R has been shown to induce ERK 1/2 activation by several mechanisms, both in human normal melanocytes and melanoma cells^19^ and in mouse melanoma cells^20^. ERK1/2 is a kinase that has been shown to phosphorylate EGFR on T669 and to inactivate the receptor^21^. In the phosphoproteomic data, EGFR T669 was the only EGFR site found to be increasingly phosphorylated by BMS-470539. This resulted in us testing if ERK 1/2 activity is regulated and involved in MC1R induced stress fiber formation and could be the kinase involved in EGFR T669 phosphorylation. At 5 min of BMS-470539 an increase in phospho-ERK 1/2 was observed (Fig. 6A). When visualizing stressfibers, it was shown that inhibiting ERK1/2 using PD98059 markedly diminished the beneficial effect of BMS-470539 on stressfibers found in PS exposed podocytes, (Fig. 6B). Calculations showed reduction of stress fiber content in PS treated cells with BMS-470539 treatment significantly increasing stress fiber content in PS treated cells. PD98059 treatment significantly diminished the beneficial rescue effect of BMS-470539 treatment, as shown in Fig. 6C. This indicates that ERK 1/2 is regulated by MC1R activation and that it has a major role in MC1R stabilization of stressfibers.

### MC1R promotes stressfibers by inhibiting EGFR signaling

**Figure 7:**
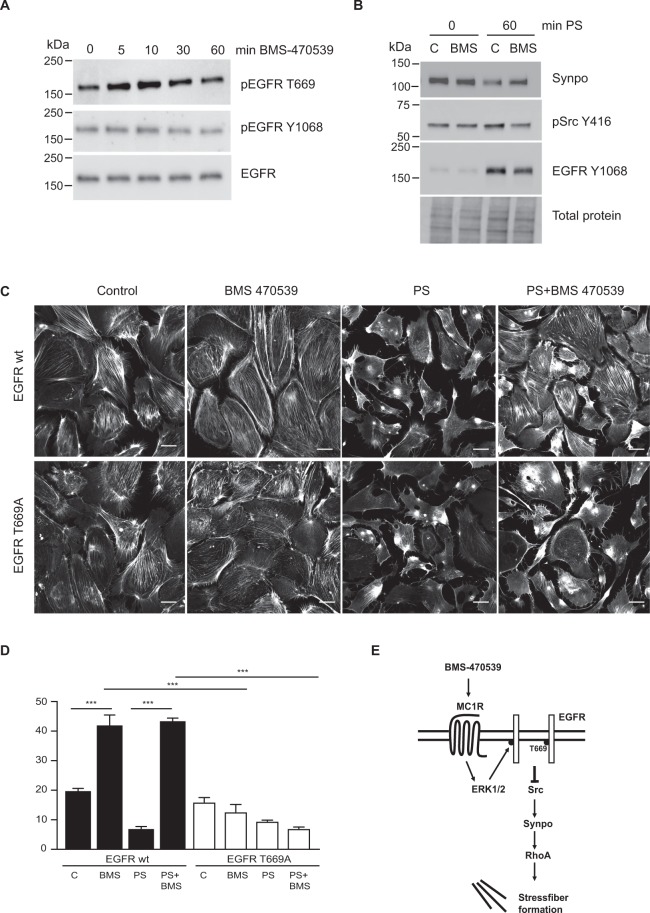
MC1R promote inhibition of EGFR dependent loss of actin stress fiber. (**A**) Western blots showing phosphorylation of EGFR on Threonine 699 (pEGFR T669) and Tyrosine 1068 (pEGFR Y1068) together with total levels of EGFR (EGFR) in podocytes treated with 0,5, 10, 30 and 60 min of BMS-470539. (**B**) Western blots on protamine sulfate treated podocytes (PS) in combination with BMS-470539 or vehicle (**C**) probed for synaptopodin (Synpo), phosphorylated Src on tyrosine 416 (pSrc Y416) and EGFR Y1068. Total protein is used as loading control. (**C**) Visualised actin stressfibers and (**D**) Actin stressfibers calculated in podoctes either overexpressing the EGFR wildtype (EGFR wt) or the point mutated T669A EGFR (EGFR T669A) treated with vehicle (**C**), protamine sulfate (PS), MC1R agonist BMS-470539 (BMS) or protamine sulfate and BMS-470539 (PS + BMS). (n = 3, ***p < 0.001, ANOVA.) Scale bar 20 µm. (**E**) MC1R stabilizes stressfibers by decreasing EGFR activation: a model. BMS-470539 activates the MC1R receptor promoting ERK 1/2 phosphorylation which phosphorylates EGFR T669 and inhibits its activation of Src. This results in stabilization of synaptopodin and RhoA induced stress fiber formation.

To further confirm the phopshoproteomic data, with MC1R induced phosphorylation of EGFR T669, western blot analysis was performed using a specific antibody detecting pT669 EGFR. After 5 to 10 min of BMS-470539 treatment an increase of pT669 EGFR was observed while no differences in phosphorylation of the activation site Y1068 in EGFR or total EGFR were detected (Fig. 7A). Activation of EGFR by PS in podocytes induces Src activation and degradation of synaptopodin and loss of stressfibers due to activation of Rac1^13^. Activation of MC1R resulted in phosphorylation of EGFR T669, reportedly being a site for inactivation of the receptor^21^, which led to the speculation that MC1R activation could promote synaptopodin stabilization by inhibiting EGFR induced Src activation. PS induced Src activation was shown to be attenuated (pSrc 416) following BMS-470539 treatment and synaptopodin levels stabilized, while EGFR activation (pEGFR Y1068) was decreased (Fig. 7B). The importance of MC1R induced phosphorylation at T669 EGFR in stress fiber formation was shown in podocytes overexpressing wt EGFR or the phosphor-resistant T669A EGFR. MC1R activation using BMS-470539 was able to restore stressfibers in PS exposed EGFR wt overexpressing cells, while its effect was not seen in T669A EGFR overexpressing cells (Fig. 7C,D). This emphasized the importence of downstream MC1R phosphorylation of the EGFR T669 site in actin cytoskeleton formation. Taken together these findings show that activation of the MC1R receptor results in ERK 1/2 dependent phosphorylation and inhibition of EGFR (pT669), leading to decreased Src activation and stabilization of synaptopodin and stress fiber formation.

## Discussion

In this study, we have taken a comprehensive approach to identify how the ACTH receptors, the MCRs, are regulated in patients with nephrotic syndrome and elucidate their effects on glomerular function by focusing on podocyte preservation. Increased glomerular expression of one of the MCRs, the MC1R, was found to be linked to nephrotic syndrome, both in patients and in the PAN rat. The MC1R regulated actin cytoskeleton signaling by inhibiting EGFR and destabilizing synaptopodin, thereby restoring podocyte stressfibers.

The fact that MC1R was found to be the most abundant ACTH receptor in the glomeruli, in combination with it being the only MCR that is augmented in nephrotic syndrome (Fig. 1A), confirm its role as being the receptor responsible for the beneficial effects of ACTH seen in patients with nephrotic syndrome. Our previous studies indicated that MC1R signaling is favorable to podocyte function and when exposed to PAN in culture, podocytes upregulated MC1R at both the mRNA and protein level^14^. Hypothetically, this might act as a response to injury, reflecting impaired glomerular function with podocytes safeguarding against foot process effacement by stabilizing the actin cytoskeleton. MC1R may be a promising therapeutic target in the treatment of nephrotic patients and thereby eliminate the unwanted side effects of ACTH, which acts via the MC2R. Podocytes exposed to the nephrotoxic agent PS increased MC1R expression (Fig. 2A) and podocytes overexpressing the MC1R responded to the MC1R agonist BMS-470539 by restoring stressfibers. MC1R E92K increased baseline levels of intracellular cAMP (Suppl Fig. 1), consistent with the results of Benned-Jensen *et al*.^22^. This Glu-to-Lys substitution at residue 92 of the MC1R was shown to induce constitutive MC1R activity, increased cAMP accumulation as well as CREB activation, which reflects long-term changes in cAMP level^22^. However, the E92K mutant does not exhibit all of the downstream MC1R effects; ERK1/2 was not constitutively activated, indicating only partial activation. Thus, overexpressing the constitutively active MC1R E92K mutant only partially protected podocytes from the effects of PS (Fig. 3A,B). When performing phosphoproteomics on podocytes overexpressing MC1R and exposing them to a MC1R specific agonist (BMS-470539), analysis of downstream signaling events revealed *actin cytoskeleton* signaling as the most significant pathway (Fig. 4). In addition, ERK1/2 was shown to be central in the top ranked affected MC1R network (Fig. 5). This highlighted ERK1/2 as being part of the main pathway downstream of MC1R in podocytes. In melanocytes, the MC1R has been shown to promote ERK1/2 activation, independently of cAMP^19^, and in podocytes we found ERK1/2 to be activated and phosphorylated after 5 to 10 min following addition of BMS-470539, while blocking ERK1/2 inhibited MC1R stress fiber formation (Fig. 6). This could explain how patients with nephrotic syndrome carrying MC1R mutations impairing cAMP response can benefit from ACTH treatment^23^, by still having intact ERK1/2 signaling downstream of the MC1R.

ERK1/2 is known to function as a negative modulator of EGFR by phosphorylating EGFR T669 leading to inactivation of the receptor^21^. In the phosphoproteomic data we found T669 in EGFR to be phosphorylated downstream of the MC1R (Fig. 5). In addition, our previous studies showed that EGFR activation promotes destabilization of synaptopodin through Src with loss of stressfibers in podocytes due to increased Rac1 activation^13^. Taken together, this could explain how MC1R activation promotes stressfibers in podocytes by inhibiting EGFR signaling. MC1R promoted EGFR inhibition by phosphorylation at T669 was shown to be central to actin stabilization, since podocytes overexpressing a phospho-resistant EGFR T669A did not promote actin cytoskeleton formation when exposed to BMS-470539 (Fig. 7C-D).

In summary, this study proposes MC1R as being responsible for the beneficial effects of ACTH on the glomerular filtration barrier in patients with nephrotic syndrome. This is mediated through downstream activation of ERK 1/2 and its negative feedback phosphorylation (T669 EGFR) and inhibition of EGFR activity, thereby inhibiting Src activity and stabilization of synaptopodin promoting RhoA stress fiber formation in podocytes (Fig. 7E).

## Methods

### Microarray analysis of renal biopsy data from FSGS and MN patients

Public data with GEO (https://www.ncbi.nlm.nih.gov/geo/) accession number GSE47183, GSE32591 and GSE37460 were used for analysis. The samples were experimented at 5 different time points on two different platforms (Affymetrix Human Genome U133 plus 2.0 and Affymetrix Human Genome U133A). Data processing was done in R (University of Auckland, Auckland, New Zealand). The CEL raw data were re-downloaded and normalized using the Robust Multiarray Averaging algorithm with quantile normalization method in individual batches based on the latest custom chip definition file (CDF file, version 22; BrainArray, Micro- array Laboratory, Ann Arbor, MI). The sample quality was checked using a selection of plots: normalized unscaled SEM plot, a relative log expression plot, an RNA degradation plot, and a principal component analysis plot. A sample was considered an outlier if more than three of four plots showed bad quality. Since the microarray experiments were done on two platforms, normalized data in each batch were aligned and matched with the same probes. Outliers were excluded from the analysis, and the normalized data from each batch were then merged using the empirical Bayes algorithm^24^. Batch corrected data were clustered using the hierarchical Ward averaging method^25^ and principal coordinate analysis (PCA). Samples that showed bad quality on clustering and PCA plot were excluded. The data were then statistically analyzed using Significant Analysis of Microarray (SAM)^26^ as implemented in MultiExperiment Viewer (MeV; Dana-Farber Cancer Institute, Boston, MA). Significantly regulated genes were selected with SAM q value < 0.05.

### Puromycin aminonucleoside nephropathy (PAN) rat and *In-situ* Hybridization

All animal studies were conducted in accordance with IACUC protocols and guidelines and approved by the Mallinckrodt Pharmaceuticals Institutional Animal Care and Use committee. Female Sprague-Dawley between 200–275 g were received and allowed to acclimate for a minimum of 48 hours before study initiation. Female Sprague-Dawley between 200–275 g were placed in metabolism cages on day-(−1) and acclimated for 24 hours, followed by a 24 hour urine and blood draw for baseline on day-0. On day-0 a single IV dose of PAN (50 mg/kg) was administered, followed by booster doses of PAN (20 mg/kg) on day-14, 21, and 28. All animals were sacrificed on day-56 and kidneys were removed and fixed in 10% NBF for 24–48 hours, then processed to paraffin blocks (FFPE).

*In-situ* hybridization (ISH) was performed using Advanced Cell Diagnostics (ACD) Inc© RNAscope chromogenic hybridization platform. Kidney tissue sections were hybridized with a DNA probe specific for rat Mc1r mRNA (ACD, Cat# 474469) and detected using conjugated HPR enzymes, on a branched DNA backbone as part of the RNAscope detection kit, and visualized with DAB substrate and hematoxylin counterstaining. Whole slide tissue sections were scanned with the Nanozoomer S210 digital slide scanner (Hamamatsu Photonics K.K). Approximately 100 glomeruli for each right and left kidney were outlined and detection of Mc1r(+) positive cells were analyzed with Visiopharm quantitative image analysis software.

### Podocyte cell culture and transient transfection

Culture of conditionally immortalized mouse podocytes was performed according to previously described protocols^27^. The cells were differentiated for at least 7 days before infection with lentivirus and subsequent experiments according to previously published methods^14^. Podocytes were treated with 5,6 ng/ml BMS-470539 (Bristol-Myers Squibb), 600 μg/ml protamine sulphate and 2,7 ng/ml PD98059 (Sigma Aldrich).

### Plasmid constructs and lentiviral overexpression

The cDNA coding for wild-type mouse MC1R and the murine E92K MC1R mutant^22^ was cloned from the pcDNA3.1+ vectors as NheI – KpnI fragments into the VVPW-EGFP vector. To obtain VVPW-Cherry MC1R construct, the EGFP-sequence was substituted by the coding sequence for mCherry using the BamHI and NotI sites. The wild-type human EGFR and mutated EGFR T669A cDNA was cloned out of the pHEX-vectors as NheI-BsrGI fragments into the VVPW-EGFP vector obtaining a pyromycin resistant gene. A VVPW-LifeAct-GFP vector was used for the LifeAct experiments^15^. A list of all vectors used is found in supplement Table 1. The lentivirus-mediated protein overexpression method was performed as previously described^14,27^. The cells were used in further experiments at least 4 days after virus transduction. For the experiments with the EGFR construct, the undifferentiated cells were infected with the appropriate EGFR construct followed by selection with puromycin (1μg/ml). On day 7, post differentiation the podocytes were infected with MC1R-EGFP and the cells were used in further experiments and processed for immunostaining 7 days post the last infection.

### LifeAct

For LifeAct experiments, podocytes overexpressing LifeAct-GFP, MC1R-mCherry or E92K-MC1R-mCherry were used. The cells were placed on a Zeiss Confocal LSM700 Microscope (Carl Zeiss AB) and a total of 120 images were acquired with 30 sec intervals during 60 min using an x 40 lens and Zeiss ZEN Black software (Carl Zeiss AB). The images from the time-lapse series were exported and analyzed using computer-assisted image analysis with software from Visiopharm (Visiopharm). Visiopharm was used to analyze the photographs for calculation of the total podocyte area over time and compared to the 0-min time point.

### MS analysis and pathway analysis

Proteins were digested using the filter-aided sample preparation (FASP)^28^. 500 µg protein amount per sample were used for the phospho-enrichment. The Orbitrap Fusion Tribrid mass spectrometer interfaced to an Easy-nLC1000 was used to analyze the TMT labeled samples. Briefly: phosphopeptides were enriched using Pierce™ TiO_2_ Phosphopeptide Enrichment and Clean-Up Kit (Thermo Fisher Scientific), the resulting eluates evaporated in a vacuum centrifuge and subjected to isobaric mass tagging reagent 6-plex TMT® (Thermo Fisher Scientific), and finally desalted using Pierce™ C18 spin columns (Thermo Fisher Scientific). Further separation of the TMT labeled peptides for total protein analysis was performed with Strong Cation Exchange Chromatography (SCX). MS raw data files for each TMT set were matched for identification and relative protein quantification using Proteome Discoverer version 1.4. The networks analyses were generated through the use of IPA (QIAGEN Inc., https://www.qiagenbioinformatics.com/products/ingenuity-pathway-analysis) using previous described algorithms for the use of IPA^29^. The mass spectrometry proteomics data have been deposited to the ProteomeXchange Consortium via the PRIDE^30^ partner repository with the dataset identifier PXD009198.

### Melanocortin Receptor mRNA Expression Analysis using Droplet Digital PCR

mRNA was purified, CDNA prepared and mRNA expression analysed using TaqMan Fam labeled probes, see Supplement Table 2. Droplets were made using the Biorad QX200 Droplet Generator and analyzed on the QX200 droplet reader with QuantaSoft Software.

### SDS page and Western Blot

SDS-page and western blotting were performed according to the V3 Western Workflow suggested by the manufacturer^31^. The membranes were blocked for 1 h and incubated with primary antibody and secondary antibodies (Supplement Table 3). Bands were visualized using Clarity Western ECL and developed in the ChemiDoc Touch with bands being normalized against total protein detected using the stain free protocol protocol in the BioRads 3 V Western workflow using the Image Lab Software (Bio-Rad Laboratories).

### Immunocytochemistry and stress fiber calculations

Immunocytochemistry and stress fiber quantification was performed according to previously published methods^27^. The antibodies used are presented in (Supplement Table 3). Images were obtained on a Zeiss confocal microscope.

The Stress fiber quantification was performed according to previously published methods^27^. The experiments were repeated in at least triplicates where at least 50 cells were counted in each experiment from 6–10 randomly chosen images.

### Statistical analysis

GraphPad Prism 6 software was used to perform statistical analyses. Differences between groups were compared with a Student’s T-test or one-way ANOVA when comparing more than 2 groups. P < 0.05 was considered statistically significant. Data from the IPA software was analyzed with Fisher’s exact test, P < 0.001 was considered statistically significant. All data are presented as mean ± SD.

### Disclosure

The studies were partly supported by Mallinckrodt Pharmaceuticals. LBu has received compensation as a member of the scientific advisory board of Mallinckrodt Pharmaceuticals. Apart from that there is nothing to disclose.

## Electronic supplementary material

Supplementary Information
