# Supplementary material for: Amplification of the Melanocortin-1 Receptor in Nephrotic Syndrome Identifies a Target for Podocyte Cytoskeleton Stabilization
Source: Sci Rep. 2018 Oct 24;8:15731. doi: 10.1038/s41598-018-34004-7 (PMC6200758; doi:10.1038/s41598-018-34004-7)

Amplification of the Melanocortin-1 Receptor in Nephrotic Syndrome Identifies a Target for Podocyte Cytoskeleton Stabilization

Lovisa Bergwall MD[1], Hanna Wallentin PhD[1], Johannes Elvin PhD[2], Peidi Liu PhD[1], Roberto Boi PhD[1], Carina Sihlbom PhD[3], Kyle Hayes MS[4], Dale Wright PhD[4], Börje Haraldsson MD, PhD[1], Jenny Nyström PhD[1] and Lisa Buvall PhD[1*]

[1]Department of Physiology, Institute of Neuroscience and Physiology, [2]Department of Molecular and Clinical Medicine, [3]The Proteomics Core Facility at the Institute of Medicine at the Sahlgrenska Academy, University of Gothenburg, Sweden. [4]Mallinckrodt Pharmaceuticals, Hazelwood, Missouri, USA

SUPPLEMENT MATERIAL

Table of contents **Page**

Supplement figure 1 2
cAMP response in MC1R overexpressing podocytes

Supplemental table 1-3 3
1. Vectors used for virus overexpression in cultured podocytes
2. TaqMan Fam labeled probes for mRNA analysis
3. Antibodies

Supplement table 4 4-5
Ingenuity Canonical Pathways identified in BMS-470539 treated podocytes

Full Western Blot membranes 5-9

Information to access to ProteomeXchange via the PRIDE database:

Project Name: MC1R stabilize the actin cytoskeleton
Project accession: PXD009198
Username: reviewer40041@ebi.ac.uk
Password: R8wV8tyC

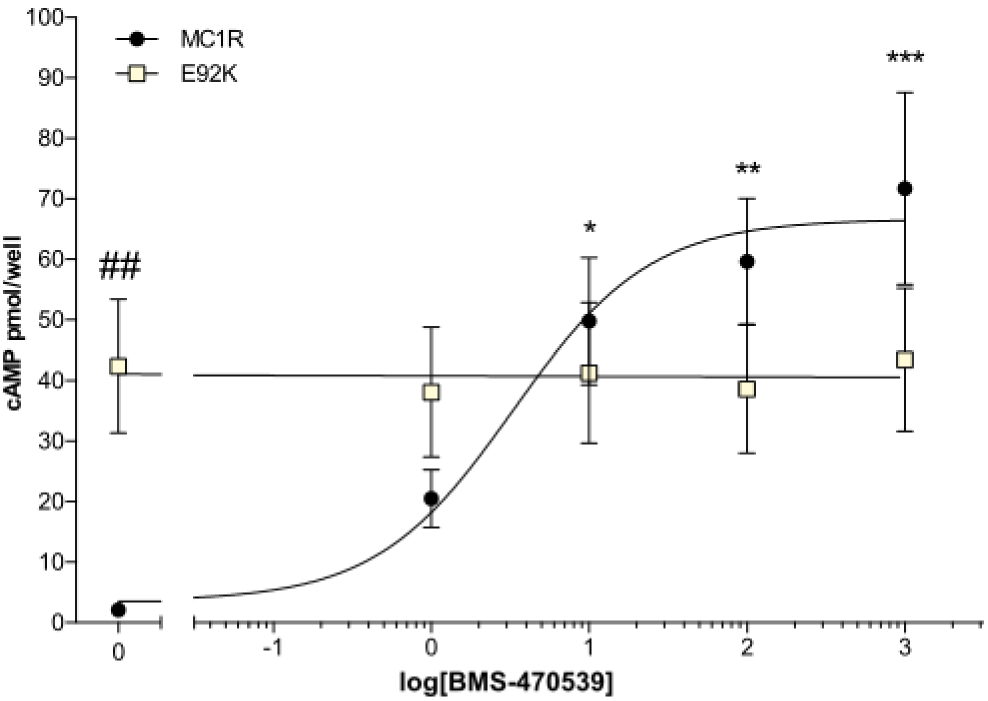

Supplement figure 1. cAMP response in MC1R overexpressing podocytes.

The cAMP levels of podocytes overexpressing MC1R and MC1R-E92K were examined after stimulation for 30 min with BMS-470539. The intracellular cAMP levels in the MC1R podocytes were dose-dependently elevated 30-fold (from 2.07 ± 0.26 to 71.70 ± 15.89 pmol/well, 1000 nM BMS-470539). The MC1R mutant E92K had significantly higher baseline level of intracellular cAMP, 20 times higher than the MC1R overexpressing podocytes (42.38 ± 11.00 pmol/well), which is ~60 % of the maximal response to BMS-470539 in the MC1R overexpressing podocytes. The MC1R mutant E92K did not respond with cAMP increase to stimulation with BMS-470539. MC1R podocytes responds dose-dependently to BMS-470539 with significantly increased intracellular cAMP accumulation after stimulation with BMS-470539 for 30 min (●), n = 6, ANOVA, *P < 0.05, **P < 0.01, ***P < 0.001. Podocytes overexpressing the constitutively active E92K MC1R mutant (□) has significantly elevated cAMP levels even without MC1R stimulation (compared to MC1R overexpressing cells, n = 6, Student's T-test, ##P < 0.01) and are not affected by addition of BMS-470539. Values are presented as pmol/well ± s.e.m.

Supplemental Table 1: Vectors used for virus overexpression in cultured podocytes

Vector
VVPW-EGFP
VVPW-MC1R-EGFP
VVPW-MC1R-E92K-EGFP
VVPW-mCherry
VVPW-MC1R-mCherry
VVPW-MC1R-E92K-mCherry
VVPW-LifeAct-GFP
VVPW-EGFR
VVPW-EGFR T669A

Supplemental Table 2: TaqMan Fam labeled probes for mRNA anaysis

Vector	ref#
MC1R	Mm00434851 Applied Biosystems
MC2R	Mm00434865 Applied Biosystems
MC3R	Mm00434876 Applied Biosystems
MC4R	Mm00457483 Applied Biosystems
MC5R	Mm00442970 Applied Biosystems
Hex labeled endogenous control Rplp0	dMmuCPE5195429 Biorad

Supplemental Table 3: Antibodies

Vector	ref#	Dilution
EGF receptor	4267, Cell Signaling Technology	1:1000
p-EGF receptor T669	3056, Cell Signaling Technology	1:1000
p-EGF Receptor Y1068	3777, Cell Signaling Technology	1:1000
p-ERK1/2	9101, Cell Signaling Technology	1:1000
ERK	9102, Cell Signaling Technology	1:1000
Src	2123, Cell Signaling Technology	1:1000
p-Src Y416	6943, Cell Signaling Technology	1:1000
synaptopodin NT	Gift from Dr Peter Mundel, MA, Boston	1:5000
Anti-rabbit secondary antibody IgG HRP conjugate	W401B, Promega	1:10000
Rhodamine Phalloidin	R415, Life Technologies	1:750
MC1R	Pierce Technologies	1:100
Alexa Fluor 488 goat anti-rabbit IgG	A11034, Life Technologies	1:2000

Supplement table 4. Ingenuity Canonical Pathways identified in BMS-470539 treated podocytes

5 min BMS				
**Ingenuity Canonical Pathways**	**-log(p-value)**	**Ratio**	**z-score**	**Molecules**
Actin Cytoskeleton Signaling	5.81E00	5.48E-02	-2.309	SHC1,PXN,MPRIP,MYH9,PTPN11,PPP1R12A,FLNA,ARHGEF7,PAK2,ARHGAP35,TRIO,MYL12A
Thrombin Signaling	4.6E00	5.03E-02	-1.897	SHC1,MPRIP,PLCE1,PTPN11,PPP1R12A,PDPK1,ARHGEF2,PRKD3,EGFR,MYL12A
Caveolar-mediated Endocytosis Signaling	4.26E00	8.7E-02	NaN	FLNB,ITSN1,FLNA,FLNC,PTRF,EGFR
Phospholipase C Signaling	4.23E00	4.55E-02	-1.667	SHC1,MPRIP,PLCE1,PPP1R12A,ARHGEF7,HDAC7,ARHGEF18,ARHGEF2,PRKD3,MYL12A
ILK Signaling	4.05E00	4.81E-02	-0.707	FLNB,PXN,MYH9,PTPN11,PPP1R12A,FLNA,FLNC,PDPK1,NACA
Integrin Signaling	3.65E00	4.27E-02	-2.646	SHC1,PXN,MPRIP,PTPN11,PPP1R12A,ARHGEF7,Wasl,PAK2,MYL12A
Protein Kinase A Signaling	3.56E00	3.23E-02	-1.667	AKAP12,AKAP2,FLNB,AKAP13,PXN,PLCE1,PTPN11,PPP1R12A,FLNA,FLNC,PRKD3,MYL12A
ErbB Signaling	3.49E00	6.32E-02	NaN	SHC1,PTPN11,PAK2,PDPK1,PRKD3,EGFR
Virus Entry via Endocytic Pathways	3.44E00	6.19E-02	NaN	FLNB,PTPN11,ITSN1,FLNA,FLNC,PRKD3
PAK Signaling	3.44E00	6.19E-02	-2.449	SHC1,PXN,PTPN11,ARHGEF7,PAK2,MYL12A

10 min BMS				
**Ingenuity Canonical Pathways**	**-log(p-value)**	**Ratio**	**z-score**	**Molecules**
PAK Signaling	2.57E00	4.12E-02	NaN	PXN,PAK4,CFL1,MYL12A
Axonal Guidance Signaling	2.33E00	1.82E-02	NaN	PLCD1,PXN,PAK4,PLCE1,CFL1,ABLIM1,EPHA2,MYL12A
Phospholipases	2.31E00	5.17E-02	NaN	PLCD1,PLA2G4A,PLCE1
Protein Kinase A Signaling	2.18E00	1.89E-02	-1.342	AKAP12,PLCD1,AKAP2,PXN,PLCE1,FLNA,MYL12A
Thrombin Signaling	2.18E00	2.51E-02	-1.342	PLCD1,PLCE1,ARHGEF2,EGFR,MYL12A
Agrin Interactions at Neuromuscular Junction	2.15E00	4.55E-02	NaN	PXN,PAK4,EGFR
Ephrin B Signaling	2.05E00	4.17E-02	NaN	PXN,CFL1,HNRNPK
Actin Cytoskeleton Signaling	2.01E00	2.28E-02	-2.236	PXN,PAK4,CFL1,FLNA,MYL12A
Phospholipase C Signaling	2.00E00	2.27E-02	-1.000	PLA2G4A,PLCE1,HDAC7,ARHGEF2,MYL12A
D-myo-inositol (1,4,5)-Trisphosphate Biosynthesis	1.95E00	7.41E-02	NaN	PLCD1,PLCE1

30 min BMS				
**Ingenuity Canonical Pathways**	**-log(p-value)**	**Ratio**	**z-score**	**Molecules**
Actin Cytoskeleton Signaling	5.18E00	4.57E-02	-3.162	SHC1,PXN,CFL1,PPP1R12A,FLNA,ARHGEF7,PAK2,TRIO,BCAR1,MYL12A
Signaling by Rho Family GTPases	4.05E00	3.75E-02	-2.121	CFL1,PPP1R12A,ARHGEF7,PAK2,VIM,ARHGEF17,ARHGEF2,SEPT2,MYL12A
PAK Signaling	4.02E00	6.19E-02	-2.236	SHC1,PXN,CFL1,ARHGEF7,PAK2,MYL12A
Phospholipase C Signaling	3.56E00	3.64E-02	-1.890	SHC1,PLCE1,PPP1R12A,ARHGEF7,HDAC7,ARHGEF17,ARHGEF2,MYL12A
RhoGDI Signaling	3.55E00	4.19E-02	1.890	CFL1,PPP1R12A,ARHGEF7,PAK2,ARHGEF17,ARHGEF2,MYL12A
Axonal Guidance Signaling	3.28E00	2.5E-02	NaN	MME,SHC1,PXN,PLCE1,CFL1,ARHGEF7,PAK2,BCAR1,ABLIM1,EPHA2,MYL12A
ILK Signaling	3.25E00	3.74E-02	0.447	FLNB,PXN,CFL1,PPP1R12A,FLNA,VIM,NACA
ERK/MAPK Signaling	3.2E00	3.66E-02	-1.890	SHC1,PXN,PPP1R12A,PAK2,BCAR1,HSPB1,EIF4EBP1
Integrin Signaling	2.95E00	3.32E-02	-2.449	SHC1,PXN,PPP1R12A,ARHGEF7,PAK2,BCAR1,MYL12A
Paxillin Signaling	2.82E00	4.55E-02	-2.236	PXN,MAPK14,ARHGEF7,PAK2,BCAR1

60 min BMS				
**Ingenuity Canonical Pathways**	**-log(p-value)**	**Ratio**	**z-score**	**Molecules**
ILK Signaling	6.04E00	5.88E-02	0.333	FLNB,MYH10,PXN,CFL1,PPP1R12A,FLNA,FLNC,VIM,PDPK1,VCL,NACA
Actin Cytoskeleton Signaling	4.57E00	4.57E-02	-3.162	SHC1,MYH10,PXN,ARHGEF12,CFL1,PPP1R12A,FLNA,ARHGEF7,VCL,SLC9A1
Thrombin Signaling	3.39E00	4.02E-02	-2.121	SHC1,ARHGEF12,PLCE1,MAPK14,PPP1R12A,PDPK1,ARHGEF2,PRKD3
Protein Kinase A Signaling	3.31E00	2.96E-02	-1.667	AKAP12,AKAP2,FLNB,MYH10,PXN,PLCE1,PPP1R12A,FLNA,FLNC,PRKD3,ANAPC1
Axonal Guidance Signaling	3.24E00	2.73E-02	NaN	MME,SHC1,PXN,ARHGEF12,PLCE1,CFL1,ARHGEF7,Wasl,RTN4,PRKD3,ABLIM1,EPHA2
Aldosterone Signaling in Epithelial Cells	3.21E00	4.32E-02	-2.000	PLCE1,DNAJC5,HSP90AB1,HSP90AA1,PDPK1,PRKD3,SLC9A1
Phospholipase C Signaling	3.1E00	3.64E-02	-1.890	SHC1,ARHGEF12,PLCE1,PPP1R12A,ARHGEF7,HDAC7,ARHGEF2,PRKD3
Neuregulin Signaling	3,00E00	5.81E-02	NaN	SHC1,HSP90AB1,HSP90AA1,PDPK1,PRKD3
D-myo-inositol (1,4,5)-Trisphosphate Biosynthesis	2.77E00	1.11E-01	NaN	PLCE1,PI4K2A,PI4KB
Leukocyte Extravasation Signaling	2.63E00	3.43E-02	NaN	PXN,MAPK14,Wasl,CTNNA1,ARHGAP12,VCL,PRKD3

Western blot full membranes:

Fig 2B:

MC1R **Total protein**

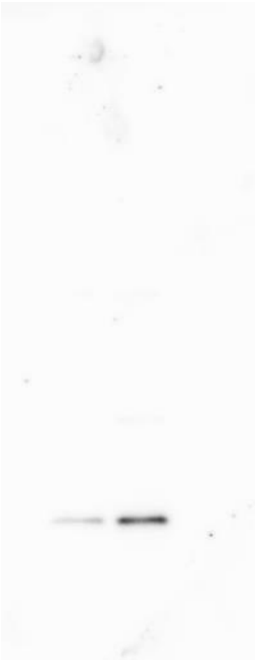

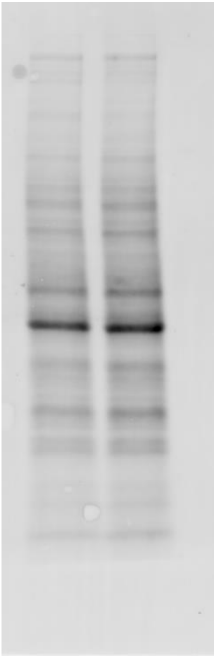

Figure 6A:

p- ERK 1/2 **Total ERK 1/2** **Total protein**

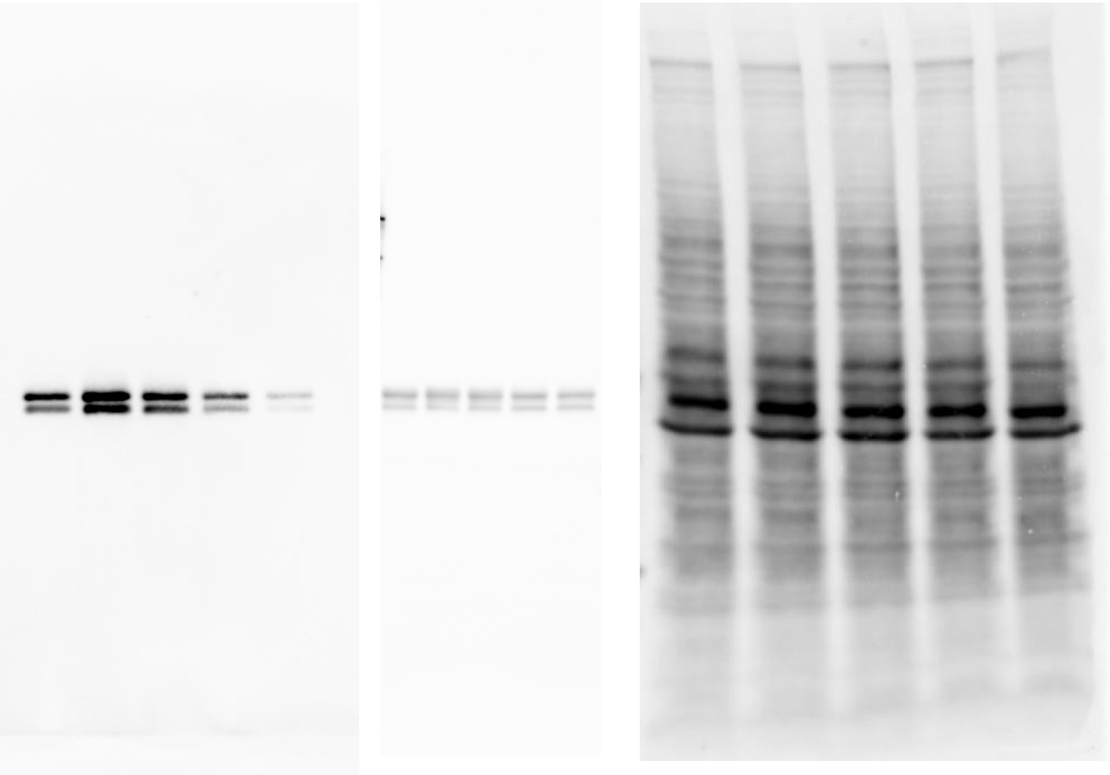

Figure 7A:

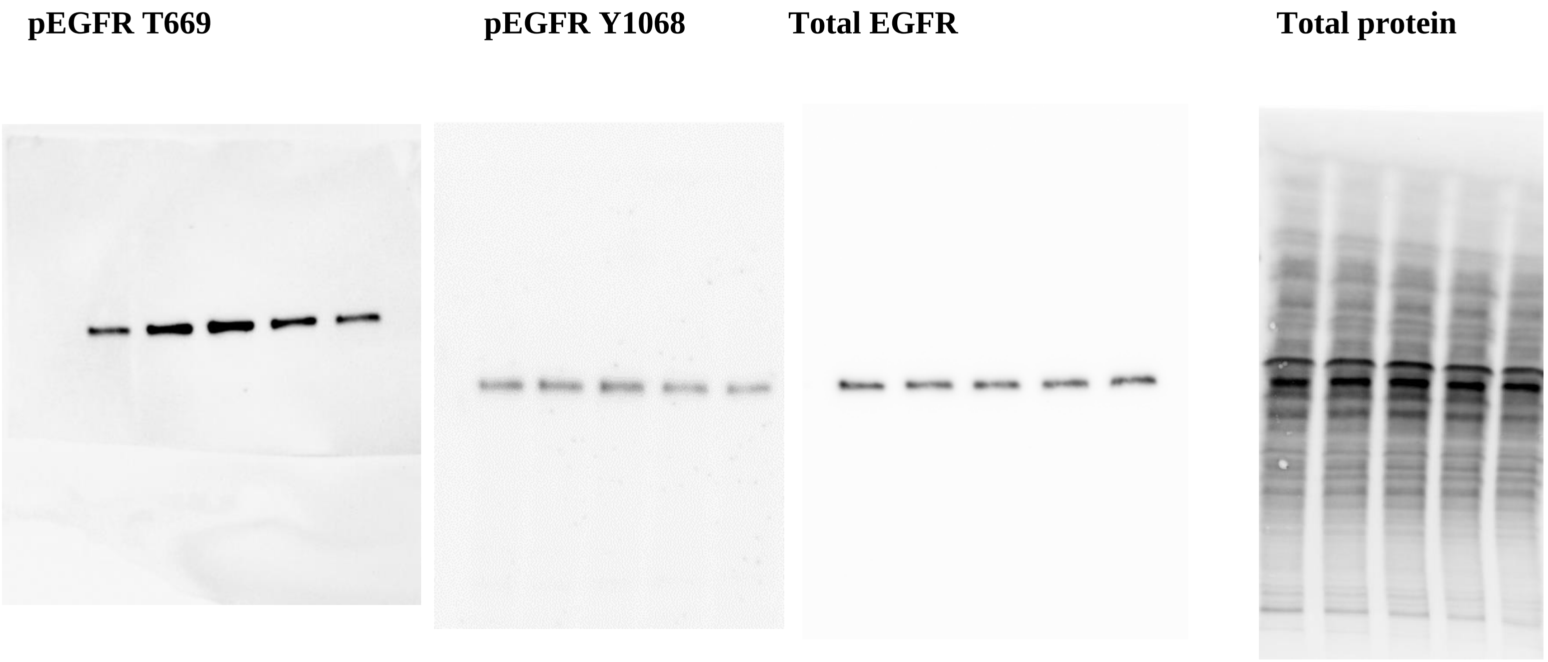

Figure 7B:

Synpo **pSrc Y416** **pEGFR Y1068** **Total protein**

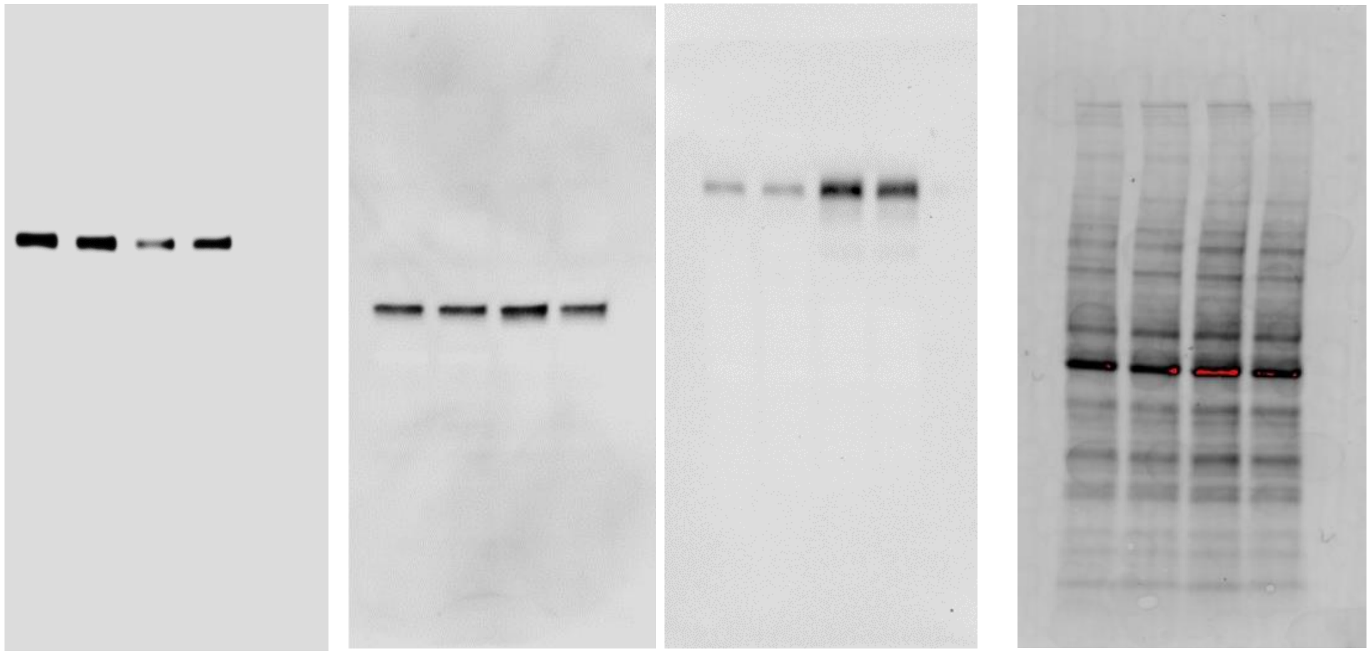

Supplement: Supplementary file 1 — Supplementary Information [file 41598_2018_34004_MOESM1_ESM.pdf]
